# Maintaining health-system functionality in response to the surge of COVID-19 cases due to the Omicron variant, Japan

**DOI:** 10.5365/wpsar.2023.14.5.1048

**Published:** 2024-02-06

**Authors:** Yuki Moriyama, Saho Takaya, Takeshi Nishijima, Howard L Sobel, Norio Ohmagari

**Affiliations:** a Disease Control and Prevention Center, National Center for Global Health and Medicine, Tokyo, Japan.; b Emerging and Reemerging Infectious Diseases, National Center for Global Health and Medicine, Graduate School of Medicine, Tohoku University, Sendai, Japan.; c World Health Organization Regional Office for the Western Pacific, Manila, Philippines.; * These authors contributed equally to this manuscript.

## Abstract

**Problem:**

The Omicron variant of severe acute respiratory syndrome coronavirus 2 caused the largest surge of coronavirus disease (COVID-19) cases in Japan starting in the summer of 2022. We describe the mechanisms introduced to provide appropriate health care to all Omicron cases, provide appropriate health care to all non-COVID-19 patients, and protect health-care workers (HCWs) while providing necessary health services. Optimization of care for elderly patients was particularly important.

**Context:**

Japan is home to 125 million people, of whom 28.6% are 65 years or older. Between January and June 2022, the country experienced 4.3 times more COVID-19 cases than in the previous 2 years (7.3 million vs 1.7 million).

**Action:**

To adjust care pathways, inpatient treatment capacity was increased, a home-based care system was established, and an on-site treatment scheme at long-term care facilities was started. Among essential health services, disruption of emergency care became most noticeable. Administrative and financial support was provided to hospitals with emergency departments to maintain emergency medical services. To protect HCWs while maintaining hospital services, flexible exemptions were introduced to enable those who became close contacts to return to work, and broadly targeted contact tracing and testing in case of nosocomial outbreaks were all helpful.

**Outcome:**

As a result of the adjustments made to inpatient capacity and patient flow, bed occupancy for COVID-19 patients decreased, mostly because many patients were cared for at home or in temporary-care facilities.

**Discussion:**

From this study, we extracted two essential lessons to aid in current and future health emergencies: how to balance the provision of acute medical care for elderly patients and maintain their well-being; and how to maintain essential health services.

## PROBLEM

**Fig. 1:**
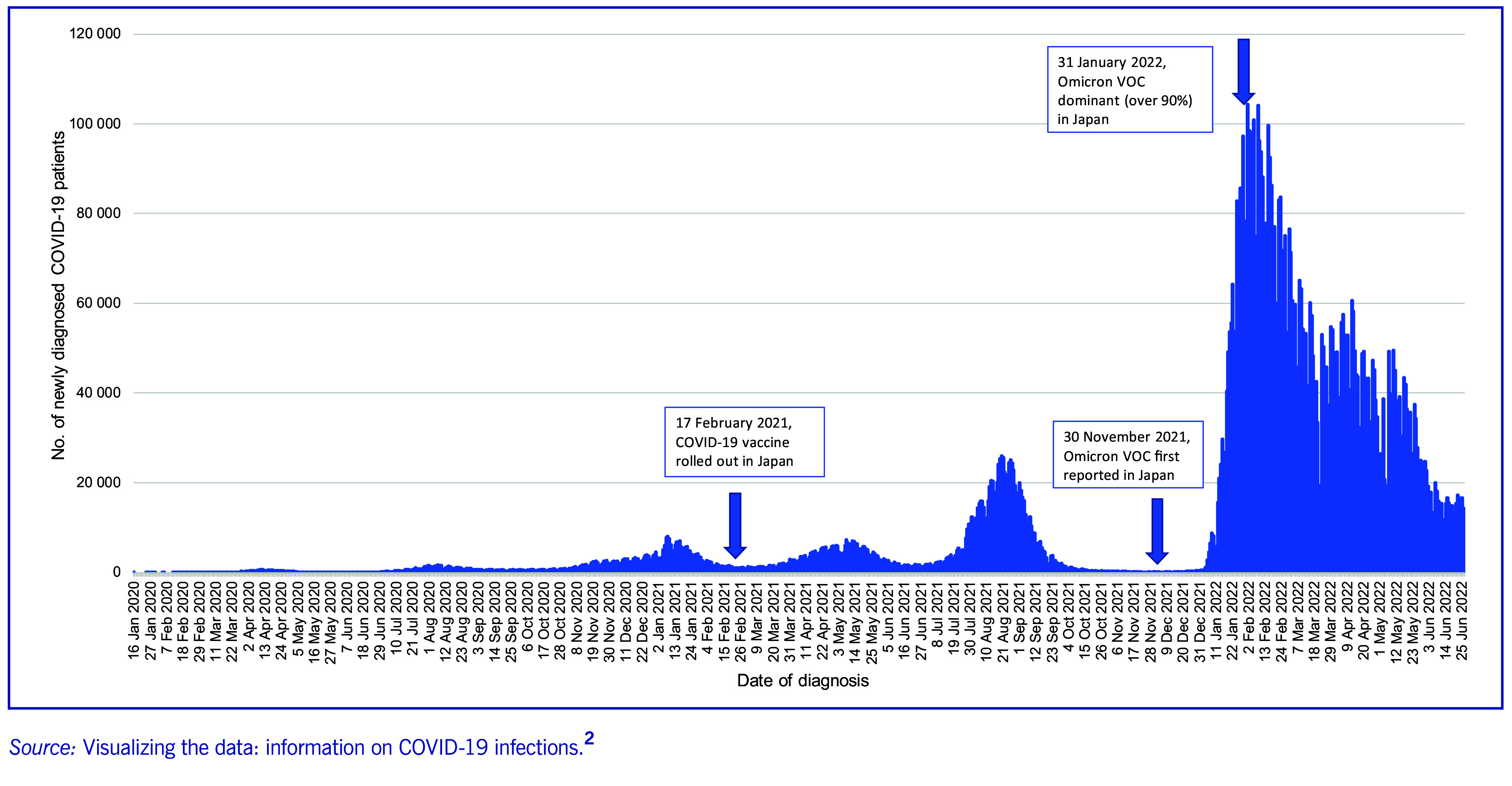
Daily new confirmed COVID-19 cases in Japan, from 16 January 2020 to 31 June 2022

The Omicron variant of concern (VOC) of severe acute respiratory syndrome coronavirus 2 (SARS-CoV-2) was first reported in Japan on 30 November 2021, and quickly spread throughout the country. As of 31 January 2022, Omicron accounted for more than 90% of all SARS-CoV-2 cases (*1*) and caused the sixth wave of coronavirus disease (COVID-19) in Japan (**Fig. 1**). (*2*) Although Omicron had decreased risk of severe disease, (*3*) its higher transmissibility resulted in the largest surge of cases to date. The number of COVID-19 cases reported in the first 6 months after Omicron began circulating in Japan was 4.3 times higher than the total number of COVID-19 cases between January 2020 and November 2021, with 7.3 million cases compared to 1.7 million reported in the previous 2 years. (*2*)

[**insert** Figure 1]

The unprecedented number of COVID-19 cases challenged the medical system in Japan, which provides virtually all citizens with national health insurance and access to medical care. The rapid increase in COVID-19 cases meant that existing care pathways and patient flow became more challenging. Maintaining health services for non-COVID-19 conditions, an issue since the beginning of the pandemic, intensified and placed a strain on the front lines of the health-care system, such as the emergency departments. The rapid increase in COVID-19 cases also reduced the number of available health-care workers (HCWs), as a significant number either became infected with SARS-CoV-2 or were close contacts of confirmed cases. Therefore, protecting HCWs from infection while maintaining necessary health services during this time of community transmission was another challenge during the Omicron wave.

In this article, we describe the mechanisms introduced to provide appropriate health care to all Omicron cases, provide appropriate health care to all non-COVID-19 patients, and protect HCWs while providing necessary health services.

## CONTEXT

Japan is the world’s first super-aged society. In 2020, 28.6% of its 125 million population were aged 65 years or older. (*4*) This had an impact on the surge of Omicron cases, as elderly COVID-19 patients often have baseline comorbidities that require additional medical care, which tends to prolong their hospitalization, puts a significant burden on hospital staff and stagnates patient flow.

Isolation and quarantine criteria and treatment protocols changed throughout the COVID-19 response as new evidence for COVID-19 became available, (*1*) and care pathways were continually adjusted accordingly. COVID-19 was relatively well controlled in Japan until the emergence of the Omicron VOC. (*1*)

COVID-19 vaccination started in Japan on 17 February 2021, with coverage of the primary series reaching 74% by 1 December 2021, which was before the identification of the first COVID-19 case due to the Omicron VOC in the country. At that time, the third dose (booster vaccination) had not yet been initiated. (*5*)

## ACTION

### Providing appropriate health care to all Omicron cases

From January 2020, when COVID-19 began to spread in Japan, inpatient capacity was increased and a smoother patient flow was developed to manage the growing number of COVID-19 cases. Prior to the pandemic, there were 1888 beds for emerging and re-emerging infectious diseases in 411 designated hospitals nationwide. This was increased to approximately 25 000 beds by November 2021 in response to the pandemic, and further increased to 40 000 by April 2022 in response to the emergence of the Omicron VOC. (*6*) Nevertheless, the rapidly increasing demand overwhelmed bed capacity.

In February 2022, to better utilize the limited designated beds, the Ministry of Health, Labour and Welfare (MHLW) recommended discharging or transferring patients to non-acute hospitals if they did not require oxygen by day 4 of hospitalization. (*7*) This recommendation was based on evidence from a February 2022 report published by the National Hospital Organization Clinical Data Archives, which stated that it was rare (0.9%, 12/1312) for patients hospitalized for COVID-19 to require oxygen after day 4. (*8*) The MHLW cautioned that patients aged > 60 years may still need careful monitoring. (*7*)

**Fig. 2:**
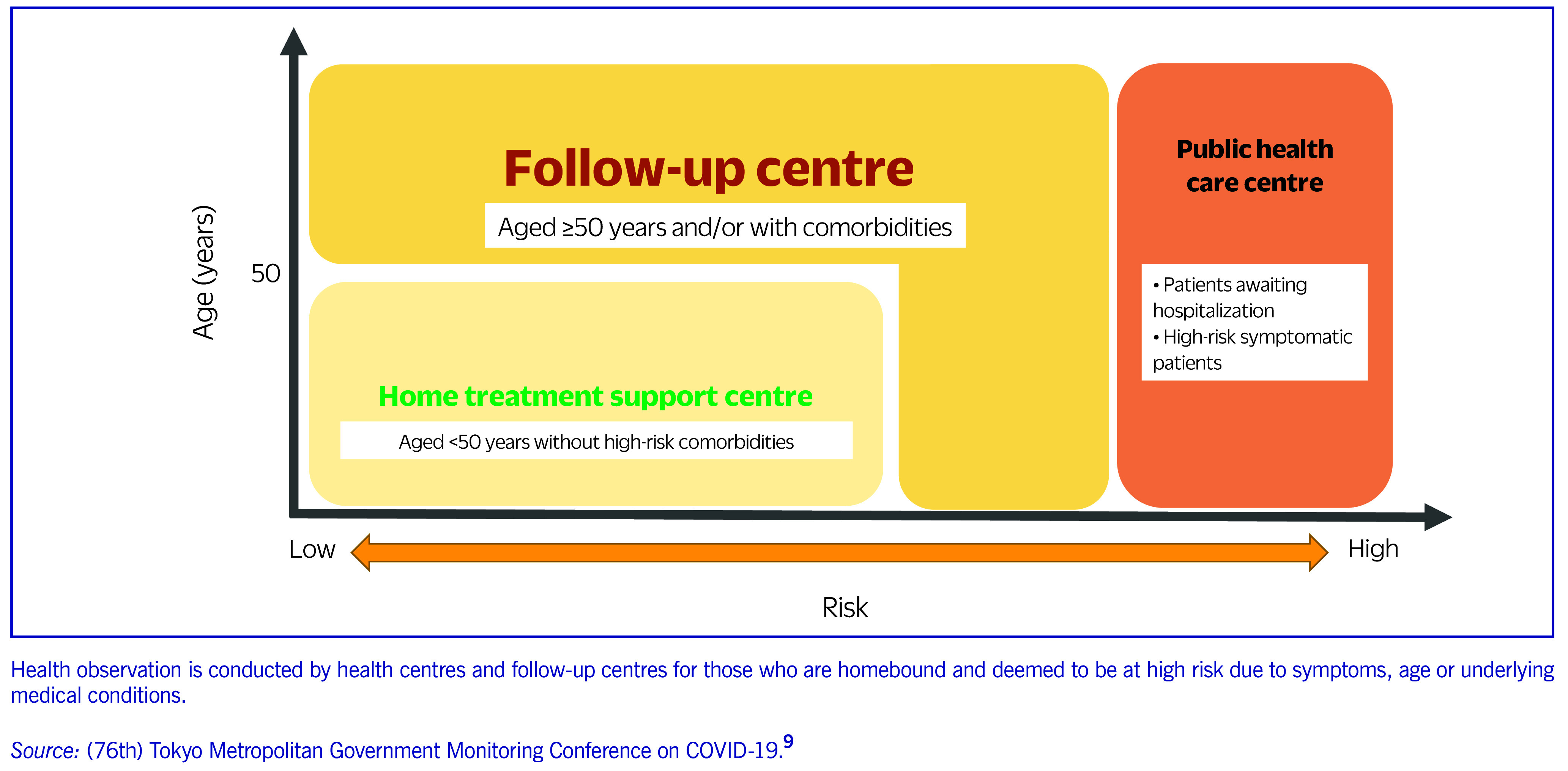
Home-based care system introduced by the Tokyo Metropolitan Government for COVID-19 patients with mild disease in Tokyo

Subsequently, a home-based care system was established to reduce the number of hospitalized cases. In January 2022, the MHLW instructed local governments to distribute pulse oximeters to patients at home, promote the use of an online self-reporting system, and establish follow-up centres for patients at risk of severe disease. Although most patients with the Omicron VOC had mild disease, sudden deterioration was possible, especially in elderly people and those with comorbidities. The home-based care system introduced by the Tokyo Metropolitan Government assessed the risk of severe disease based on age and existing comorbidities (**Fig. 2**). (*9*) The MHLW also approved presumptive diagnosis of COVID-19 without testing for people who were living with a confirmed COVID-19 case and developed symptoms suggestive of COVID-19. (*10*) Until this time, laboratory confirmation of SARS-CoV-2 infection had been compulsory for diagnosis, which led to a bottleneck in the care pathway during the Omicron wave. An on-site treatment scheme at long-term care facilities (LTCFs) was established to assist with managing COVID-19 cases. Previously, residents of LTCFs who contracted COVID-19 were transferred to acute-care facilities, even when this meant transferring a large proportion of the residents. This was because LTCFs found it difficult to implement appropriate infection prevention and control (IPC) measures. During the Omicron wave, the Tokyo Metropolitan Government began dispatching medical teams to LTCFs that requested a transfer of two or more residents with COVID-19 to local hospitals to provide medical services including monoclonal antibody therapy and antiviral drugs. This scheme spread throughout Japan, with 94% of LTCFs reporting that they had a consultation system with local doctors and nurses as of May 2022. (*11*) IPC specialists were included in the dispatch team and provided advice and training to LTCF staff.

[**insert** Figure 2]

This on-site treatment scheme at LTCFs was particularly useful due to Japan being a super-aged society. When elderly patients with COVID-19 were admitted to acute-care hospitals, it was inevitable that they were put under strict IPC measures and isolated from their family, familiar caregivers and their daily routine. This could result in decreased cognitive stimulation, physical exercise and social engagement, and cause or worsen cognitive and physical impairment. (*12*) Even outside an acute-care setting, at LTCFs and in the community, elderly people were often physically and socially isolated or distanced. Conversely, by treating patients with COVID-19 in LTCFs, elderly patients received treatment in a familiar environment. The prevention and treatment of COVID-19 in elderly people and the mitigation of its negative impact on their overall well-being needs to be well balanced.

COVID-19-designated hospitals were the main providers of COVID-19 care, with other hospitals, clinics and facilities having limited roles for COVID-19 cases. As a result, many designated hospitals became overwhelmed. Task-shifting from designated hospitals to other health-care facilities, especially the provision of care for patients with non-severe COVID-19 disease, was used to decrease the burden on designated hospitals. In June 2022, to accelerate the task-shifting, the MHLW required simpler IPC measures for health-care and nursing settings that were providing care to COVID-19 cases. (*13*) These measures specified that: COVID-19 patients could be cared for in facilities with appropriate zoning without dedicated wards; excessive environmental disinfection would be risk-based and target high-touch surfaces; and droplet and aerosol precautions would be prioritized and contact precautions could be minimized.

### Maintaining health services for non-COVID-19 conditions

The COVID-19 pandemic had a wide impact on health services, which was further exacerbated during the Omicron wave. For example, the number of difficult transport cases, when paramedics had to call more than four hospitals or spend longer than 30 minutes identifying a hospital to which they could take their patients, (*14*) was used as a proxy measure of health-care availability. Between mid-January and early March 2022, the number of difficult transport cases was approximately five times higher than during the pre-pandemic era (3417 between 20 January and 9 February 2020, compared with 15 722 between 17 January and 6 February 2022), with two thirds of these patients needing non-COVID-19 medical care. (*14*)

Many health-care facilities had to accept patients who were potentially infected with SARS-CoV-2, which required extra space, materials and human resources for IPC measures. This resulted in emergency departments receiving fewer patients despite functioning at full capacity. In late January 2022, the MHLW published a plan to maintain emergency medical services for non-COVID-19 patients in medical institutions with emergency departments. This plan included financial support to set up medical tents and portable container units to expand hospital space. Temporary accommodation was also established for COVID-19 patients waiting to be hospitalized. (*15*)

### Protecting health-care workers

Many HCWs had to take leave from work either due to their positive COVID-19 status or their close contact with a confirmed case. This resulted in fewer staff in health-care institutions, which again was exacerbated during the Omicron wave. In January 2022, the MHLW declared that HCWs who were close contacts could continue working, provided that they had completed a primary vaccination series and had a negative daily rapid antigen test for COVID-19. When a HCW became a positive case, rapid contact tracing was important to minimize further exposure of HCWs.

As the Omicron VOC had a shorter serial interval than the previous SARS-CoV-2 variants, outbreaks had often spread beyond identified contacts at the time of investigation. (*16*) This was a change from the nosocomial outbreaks due to previous SARS-CoV-2 variants or seasonal influenza virus and, therefore, initial screening for nosocomial outbreaks due to the Omicron VOC needed to be broadly targeted. To prevent the introduction of SARS-CoV-2 by patients with minor symptoms or asymptomatic infection, many hospitals implemented testing for COVID-19 for patients at the time of hospitalization.

Given the importance for health-care facilities to have a business continuity plan during the pandemic, the MHLW requested that the National Center for Global Health and Medicine publish guidance on plan development during COVID-19 outbreaks to maintain essential health services. (*17*)

## OUTCOME

**Fig. 3:**
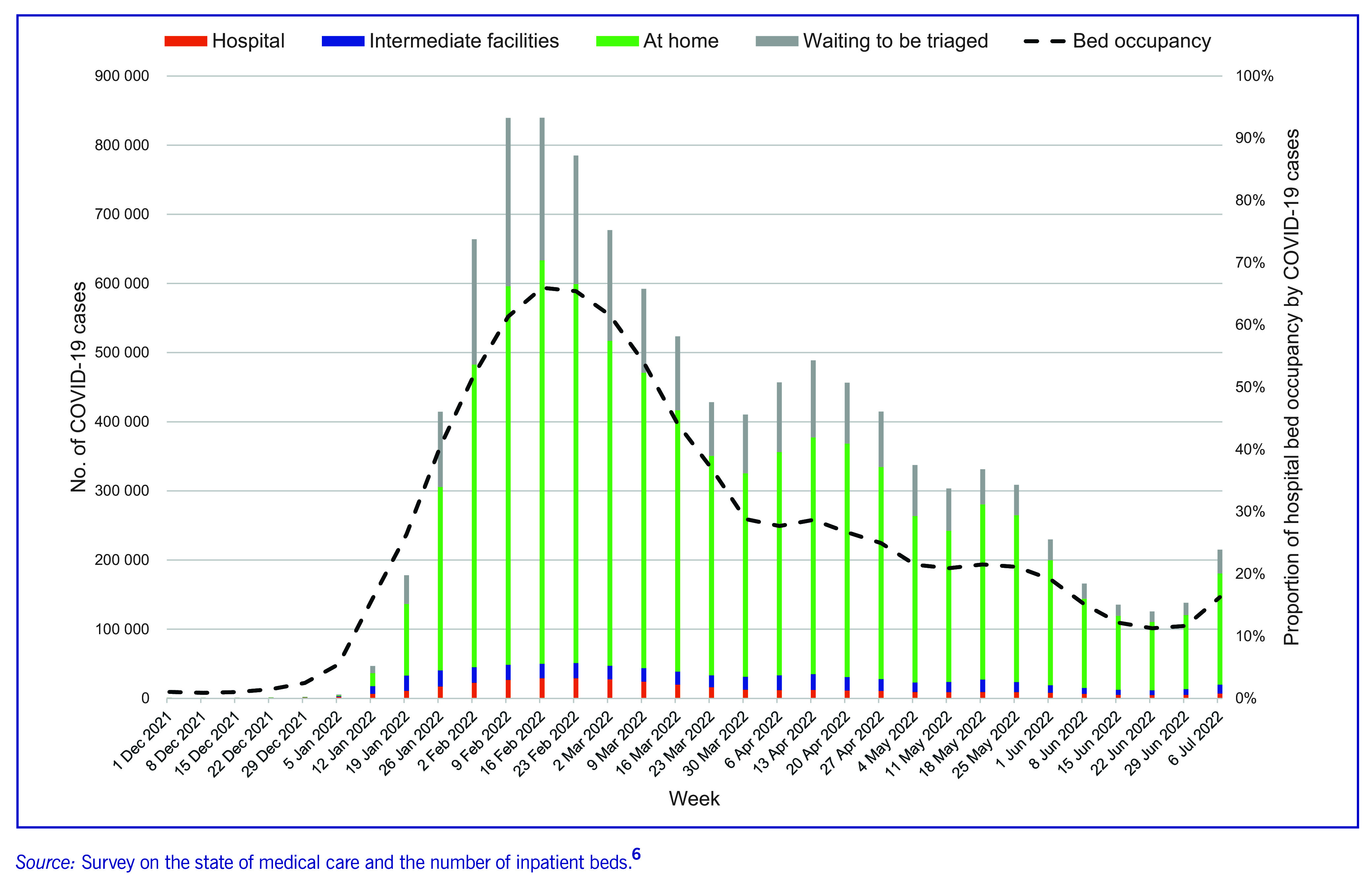
Number of COVID-19 cases in Japan by location of treatment and bed occupancy of COVID-19 cases, from 1 December 2021 to 6 July 2022

As a result of the adjustments made to inpatient capacity and patient flow, bed occupancy for COVID-19 patients decreased (**Fig. 3**). (*6*) This was mostly because many patients were cared for at home or in intermediate facilities (**Fig. 3**).

[**insert** Figure 3]

## Discussion

The unprecedented number of COVID-19 cases during the Omicron wave disrupted Japan’s health-care system and created a variety of challenges. This is despite Japan having more acute-care beds (7.7 per 1000 persons in 2019) compared to other Organization for Economic Co-operation and Development countries. (*18*) The rapid surge of cases during the Omicron wave still resulted in many patients waiting to be hospitalized and having inpatient COVID-19 treatment provided primarily at designated hospitals, which stagnated care pathways and overburdened hospitals. In response to these challenges, bed capacity in designated hospitals was increased, and a home-based care system and on-site treatment scheme at LTCFs were established. IPC measures and policies evolved flexibly to protect HCWs while maintaining essential health services. However, although MHLW supported hospitals with emergency departments to safeguard emergency medical services, difficult transport cases were still seen nationwide.

Two important lessons were gleaned from the experience. The first is how elderly people are cared for during health emergencies, and that having on-site treatment schemes at LTCFs and in the community may be preferable to care in acute-care hospitals. A Spanish study reported a significant decline in functional, cognitive and nutritional status in elderly nursing home residents regardless of infection status during the early phase of the COVID-19 pandemic. (*12*) Maintenance of a holistic quality of life needs to be incorporated into clinical management plans, as well balanced care benefits are not just for the infected but for the entire elderly population.

The second lesson is how to maintain essential health services during health emergencies. When designated COVID-19 hospitals became overwhelmed, task-shifting was important to distribute the burden to non-designated hospitals, clinics and other health-care facilities. Also, adjusting care pathways, such as home-based care for asymptomatic or mild cases and presumptive COVID-19 diagnosis of those living with confirmed cases, reduced the burden on designated hospitals and contributed to maintaining essential health services, including emergency medical care for patients with acute life-threatening conditions.

The COVID-19 pandemic will not be the last health emergency. It is crucial to prepare for the next pandemic using the lessons from the current one. This includes:

adjusting patient-care pathways when the number of patients increases and controlling patient flow as circumstances change and new scientific evidence emerges;
ensuring that care continues to be provided to patients with other diseases, especially those who require emergency care;
preventing the spread of infection in hospitals through IPC measures; and
maintaining the HCW workforce through appropriate policies.

Each of these actions needs to be tailored to the infectious disease and the evidence as it develops during the outbreak or pandemic. Japan’s experience in calibrating care pathways in their super-aged society holds valuable lessons that can benefit other countries.
